# Working with young peer researchers in sexuality studies: benefits, challenges and lessons learnt

**DOI:** 10.1080/26410397.2022.2152550

**Published:** 2023-02-22

**Authors:** Anna Page, Marianne Cense, Miranda van Reeuwijk

**Affiliations:** Senior Researcher, Rutgers, Utrecht, Netherlands

**Keywords:** peer research, youth participation, sexuality, Indonesia, Netherlands, ethics, sexual and reproductive health and rights

## Abstract

Involvement as peer researchers provides young people with an opportunity to exercise their right to participation and can facilitate unique insights into young people’s lives, social contexts, choices and negotiations. However, evidence on the approach has to date included little in-depth discussion on the complexities presented by sexuality research. Here, engaging young people as researchers is influenced by intersecting cultural discourses, particularly regarding youth agency and sexual freedom. This article provides practice-based insights from involving young people as peer researchers within two rights-based sexuality-focused research projects in Indonesia and the Netherlands. Drawing on two contrasting cultural contexts, it explores benefits and challenges regarding youth-adult power dynamics, the taboo nature of sexuality, research quality and dissemination. Recommendations for future studies include ongoing training and capacity strengthening for peer researchers which recognise cultural and educational backgrounds, strong youth-adult partnerships creating an enabling environment for the engagement of peer researchers, careful consideration of how young people are involved and critical reflection on adult-centric views of what constitutes “academic” research.

## Résumé

Le rôle de pair chercheur donne aux jeunes l’occasion d’exercer leur droit à la participation et peut faciliter des éclairages uniques sur la vie, le contexte social, les choix et les négociations des jeunes. Néanmoins, jusqu’à présent, les données sur l’approche ont inclus peu de discussions approfondies sur les complexités présentées par la recherche sur la sexualité. Ici, la participation des jeunes comme chercheurs est influencée par des discours culturels croisés, en particulier concernant le pouvoir des jeunes et leur liberté sexuelle. Cet article donne des informations tirées de la pratique sur la participation des jeunes comme pairs chercheurs dans le cadre de deux projets de recherche fondée sur les droits et orientée vers la sexualité en Indonésie et aux Pays-Bas. S’appuyant sur deux contextes culturels contrastés, il explore les avantages et les obstacles concernant les dynamiques de pouvoir entre les jeunes et les adultes, le caractère tabou de la sexualité, la qualité et la diffusion de la recherche. Les recommandations pour de futures études comprennent des activités permanentes de formation et de renforcement des capacités pour les pairs chercheurs qui tiennent compte des environnements culturels et éducatifs, de solides partenariats jeunes-adultes de façon à créer un environnement habilitant pour la participation des pairs chercheurs, une soigneuse considération de la manière dont les jeunes participent et une réflexion critique sur les idées centrées sur l’adulte de ce que constitue une recherche « universitaire ».

## Resumen

La participación como investigadores pares ofrece a jóvenes la oportunidad de ejercer su derecho a participar y puede facilitar perspectivas únicas de la vida, los contextos sociales, las opciones y las negociaciones de las personas jóvenes. Sin embargo, hasta la fecha, la evidencia sobre el enfoque ha incluido poca discusión a fondo de las complejidades presentadas por la investigación sobre sexualidad. Aquí, la inclusión de jóvenes como investigadores es influenciada por discursos culturales interrelacionados, en particular relativos a la agencia y la libertad sexual de la juventud. Este artículo ofrece perspectivas basadas en prácticas por incluir a jóvenes como investigadores pares en dos proyectos de investigación sobre sexualidad basados en derechos en Indonesia y los Países Bajos. A raíz de dos contextos culturales contrastantes, se exploran los beneficios y retos relacionados con las dinámicas de poder entre jóvenes y adultos, la naturaleza tabú de la sexualidad, y la calidad y difusión de la investigación. Las recomendaciones para futuros estudios incluyen capacitación y fortalecimiento de capacidad continuos para investigadores pares que reconocen diferentes culturas y niveles de escolaridad, sólidas alianzas entre jóvenes y adultos para crear un entorno propicio para la participación de investigadores pares, cuidadosa consideración de cómo participa la juventud y reflexión crítica de los puntos de vista centrados en adultos sobre qué constituye investigación “académica”.

## Introduction

Sexuality is a sensitive research domain, intertwined with stigma, taboo, power structures and gendered cultural norms.^1,2^ Discussing sexuality demands trust between researcher and participant.^3^ Studies focusing on young people and sexuality can establish that trust more easily when the researcher is also a young person.^4,5^ However, the way in which young people participate in sexuality research, as researchers and participants, is influenced by intersecting cultural discourses on sexual freedom, youth-adult power relations and young people’s agency.^6,7^

Recent decades have seen a global shift towards more participatory approaches to research, including research carried out by members of the community being studied.^8^ People carrying out this research are referred to by a range of terms, including “co-researchers”, “active researchers” and “peer researchers”.^9–11^ This article focuses specifically on the involvement of young people in studies about young people, and uses the term “peer researcher” (PR). As outlined in the following sections, a range of literature has already explored in depth the benefits and challenges of youth peer research.

### Benefits of youth peer research

Involving young people as PRs is recognised as having multiple benefits for young people, research quality, and the broader community or programme.^11–14^

First, for young people themselves, engagement as PRs provides an opportunity to exercise their right to participation in matters affecting them, as enshrined in the Convention of the Rights of the Child (articles 12–13),^15^ and can make an important contribution to personal empowerment.^9,16^ Such participation enables young people to develop skills, including critical thinking, and increase self-confidence.^4,9,12,17–19^ This can have wide-reaching impacts, including future employability and helping children strengthen capacity to resist exploitation.^10^

Second, involving young people as researchers in studies about their peers can benefit research quality. PRs’ rapport with peer informants can enhance depth of data, revealing young people’s perspectives from an emic (insider) perspective.^8,11,13^ Deeper, emic insights into the lives, social contexts, choices and negotiations of young people potentially provide greater validity of findings and offer more robust contributions to knowledge.^9,18,20^

Finally, youth-adult partnerships created through peer research often serve to challenge and deconstruct prevalent norms limiting young people’s agency.^16^ In this way peer research can have long-reaching effects on the community or programme in which the research takes place. Young people’s unique insights may also increase buy-in to research results^21^ and the potential to influence policy.^22^

### Challenges of youth peer research

However, peer research is not straightforward, facing a range of challenges. Firstly, peer research studies are generally commissioned by established researchers applying academic standards to the research quality. Young people who are new to research and have had little opportunity to “learn by doing” may have difficulty meeting expectations hold by older researchers. They may face challenges collecting “quality” data (e.g. following topic guides rigidly, low levels of probing), recording data (e.g. incomplete transcriptions), in analysis (e.g. jumping from synthesised results to unevidenced recommendations based on personal interpretations),^12^ and in maintaining an objective, outsider position rather than “over-identifying” with peer participants.^18^ Recognising the different starting points of youth peer researchers, capacity strengthening including initial training and ongoing support is vital to facilitate research skill development, which will in turn support research quality. Such support requires adequate resources and effective research management.^11^

Age-related disparities of power and status affect any research involving adults and children and broader youth participation. Different understandings of “youth” and young people’s perceived capabilities affect opportunities to participate; adults may be resistant to “sharing power” with those they see as having less capacity.^5^ In contexts where cultural norms enshrine a strong power disparity of adults over children, children may rarely be involved in decision making, while powerful values of obedience and respect to adults mean children are unaccustomed to expressing their views to adults.^23^ Finally, ethical responsibility to ensure research does no harm to either researchers or participants needs particular attention when both researchers and participants are young people.

### Youth peer research in sexuality studies

The sensitive nature of sexuality as a research topic brings benefits and challenges additional to those presented by youth peer research in general. Regarding benefits, sensitive issues like sexuality can particularly benefit from a strong PR-respondent rapport.^12,20^ One can also imagine that involving the community in sexuality research may serve to deconstruct related taboos and stigma. Concerning challenges, research on such a sensitive topic may cause distress or social stigma to young people involved – both researchers and participants.^24,25^ Furthermore one has to anticipate that sexuality research respondents may mention adverse experiences or request help. PRs hence need more advanced ethical research skills, which may demand more intensive training and supervision than for a less sensitive topic. While youth peer research in sexuality studies has been touched upon in some papers, to date this specific topic has rarely been discussed in-depth.^8,13^ This article hence seeks to add to the body of evidence on youth peer research by providing specific insights on the complexities presented in sexuality studies.

Drawing on case studies from Indonesia and the Netherlands, our analysis also offers insights on how contrasting cultural contexts influence youth engagement as (sexuality) researchers. Indonesia’s dominant culture is characterised by hierarchy (including of adults over young people), conservative gender norms, strong taboos regarding sex and sexuality, and expectations of abstinence before marriage.^26–28^ Conversely, Dutch culture is viewed as liberal, egalitarian and progressive towards gender equality and sexual diversity.^29,30^ By identifying the contexts’ commonalities and differences, we aim to reach a set of considerations relevant for participatory youth research on sensitive topics in different geographical and cultural settings.

## Methodology

Rutgers is an international NGO working on sexual and reproductive health and rights (SRHR). Rutgers adopts a rights-based approach to SRHR, predicated on (young) people being holders of sexual rights. The approach expands focus beyond risks such as sexuality transmitted infections and unintended pregnancies to encompass gender norms, sexual orientation, pleasure and violence, and employs Socratic approaches to stimulate critical thinking regarding sexuality and sexual choice.^31^ For Rutgers, the rights-based approach also means supporting young people’s right to participate in matters which affect them. On this basis, the organisation has structurally engaged young people in all levels of programming, including research, since 1998. The organisation also adopts a sex-positive approach which sees sexuality not *a priori* as a problem, but as a normal part of humanity, that can contribute to wellbeing if sexual rights are met.^32^

This article is based on two case studies of youth engagement as PRs in sexuality studies carried out by Rutgers: (i) *Youth Voices*, in Indonesia and (ii) “*What does good sex education look like?”*, in the Netherlands. Both studies took a rights-based sex-positive approach, and drew on the Explore toolkit for involving young people as researchers in SRHR programmes.^14^ The toolkit allows PRs to build rapport, understand research objectives, reflect on their norms and values regarding sexuality, input on research instruments and learn research skills, particularly interviewing. The two studies were designed and undertaken separately, but both evaluated youth participation allowing for subsequent joint methodological analysis. We briefly outline each study below, before explaining how PRs’ experiences were evaluated and analysed.

### Youth Voices: Indonesia

*Study background: Youth Voices*, a qualitative participatory research, engaged young Indonesian PRs to explore how messages and expectations around gender and sexuality influence their peers (aged 18–24).^7^ * The study was carried out by Rutgers in collaboration with the Universitas Gadjah Mada (UGM) Centre for Reproductive Health, and PBKI (Indonesia Planned Parenthood Association) branches in Bali, Jawa Tengah and Lampung.^†^ It was part of a broader programme, Explore4Action, gathering evidence to inform better sexual health and education services for young people in Indonesia.
*Data collected:* Data were collected in 2018 through 86 interviews and 24 focus group discussions (FGDs) in three urban areas in Indonesia: Bandar Lampung, Denpasar and Semarang.
*Recruitment of peer researchers:* Six young people were employed as PRs. The PRs were aged 21–24 and all held Bachelor’s degrees.
*Peer researcher activities:* PRs were engaged in all stages of the research including design, data collection, analysis, write up and dissemination.
*Training and support:* The project started with a one-week residential training based on the Explore toolkit (see above), followed by a mid-project residential training on analysis and report writing. Throughout the study, PRs were supported on-site by an adult, professional researcher (Site Coordinator) who had a mentoring role. Additional remote support and supervision (email, WhatsApp and regular Skype calls) were provided throughout the study by UGM and Rutgers.
*Time and remuneration:* PRs were employed full-time by UGM and paid junior researchers’ salaries. The PRs worked on *Youth Voices* for six months, and continued to be engaged with the programme over four years (with some turnover) when they also worked on other studies.
*Ethics and consent:* The study was approved by the Medical and Health Research Ethics Committee, Faculty of Medicine, University Gadjah Mada on 26 September 2018 (Ref: KE/FK/1035/EC/2018). See Box 1 on consent procedures.

Box 1. **Consent procedures**

**Dutch study**

- *Consent procedures for participation of young people as peer researchers*

All the peer researchers signed a contract in which they declared they would participate in the study as peer researchers, handle data in the prescribed way and take all measures necessary to avoid harm to participants. In this contract their involvement in the study was described, including the training and support they would get and the evaluation afterwards. The peer researchers were all above 16, which is the age in the Netherlands that you can decide for yourself to participate in a research. The study was part of a school project all final-year students have to conduct (drafting a research, the so called profielwerkstuk).

- *Consent procedures for young people as respondents*

As the study was part of the “profielwerkstuk” (a mandatory school project), participation is seen as part of schoolwork, hence parental consent is not necessary. However, the pupils themselves were asked for written consent before they were interviewed.

- *Consent procedures for participation in the evaluation of peer research experiences*

Participating in the evaluation was part of the project and as such was introduced in the beginning of the study. Each of the training weekends was evaluated directly afterwards (for multiple purposes, also to give peer researchers the opportunity to speak out about further needs for training, whether they felt self-confident for the tasks ahead and whether they needed specific support). At the end of the project a young Rutgers staff member external to the research project explicitly asked the PR’s to cooperate in the evaluation of the project. She visited each PR team to interview them reflecting on the merits and challenges of the project for themselves.

**Indonesian study**

- *Consent procedures for participation of young people as peer researchers*

In the Indonesian study, the peer researchers were employed by Gadhja Mada University (UGM), and had employment contracts setting out their roles and responsibilities, including principles of ethical research. All the peer researchers were over the age of 18, so no parental consent was needed for their participation.

- *Consent procedures for young people as respondents*

The respondents of the part of the “Youth Voices” study on which this article is based were aged 18–24. Written informed consent was obtained from all respondents prior to data collection. As the respondents were aged 18 or over, parental consent was not collected. Informed consent including for video or audio recording of the FGD/interview was reconfirmed at the time of the group discussion or in-depth interview.

- *Consent procedures for participation in the evaluation of peer research experiences*

Data included in the paper is gathered from a series of formal and informal conversations with peer researchers and staff involved in the “Youth Voices” study. At the start of each formal data collection moment (e.g. a group discussion) the purpose of the conversation was explained – to gather experiences of everyone involved in order to evaluate the process and understand how it could be improved. For informal moments of data collection, the purpose was shared later when valuable insights were gathered.

**Figure:**
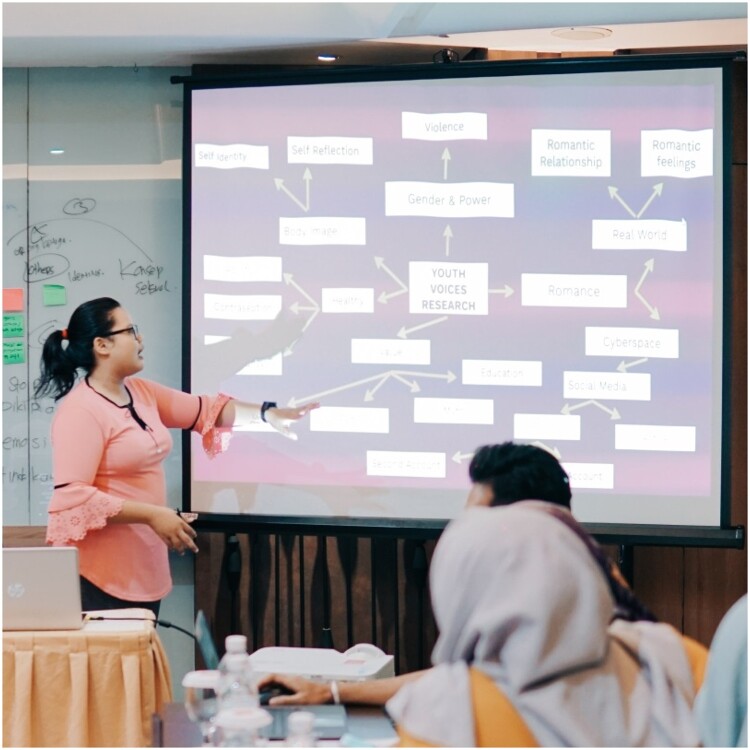
Peer researchers participating in qualitative research training in Indonesia

### What does good sex education look like: Netherlands

*Study background*: *“What does good sex education look like?”* studied the needs of young people in the Netherlands and their views on how sex education should be delivered.^3,33^ It was inspired by Rutgers’ *Sex Under 25* survey, where more than 20,000 young people (aged 12-25) rated the sexuality education they received in Dutch schools as mediocre (5.8 on a scale of one to ten).^34^ Respondents reported missing information about subjects including sexual diversity, sex in the media and sexual violence. The participatory study was designed to investigate these low ratings and understand where current practice is insufficient and should be improved.
*Data collected*: The research employed a mixed methods design, including individual interviews, FGDs and Photovoice.^‡^ 300 pupils aged 12–18 participated as respondents over six secondary schools in the Netherlands.
*Recruitment of peer researchers*: The study recruited 17 PRs (aged 16-18) with different sexuality and cultural backgrounds, across six schools. Two to four PRs were recruited per school, so they could support each other.
*Peer researcher activities*: The PRs worked closely with three adult researchers to select research methods, develop tools, collect data and carry out analysis. Researchers collected data at their own schools, across all ages and education levels. The PRs produced individual research reports and advocated for local change. They contributed to national dissemination of joint findings, delivering workshops and presenting findings in the Dutch media.
*Training and support*: The project started with an initial residential training over two weekends based on the Explore toolkit (see above), followed by mid-project residential training on data analysis and report writing. During data collection, a Rutgers supervisor visited schools to provide support and assist FGDs. The supervisor also provided follow-up communication through WhatsApp.
*Time and remuneration*: Researchers participated on a voluntary basis, in order to fulfil study requirements for their final year of secondary school. Each PR invested 80 hours in data collection data and individual report writing, two weekends training and three research group meetings.
*Ethics and consent*: The research was conducted according to Dutch legal and ethical guidelines for responsible research, including voluntary participation, safeguards against participant identity disclosure, and respect for participants.^35^ See Box 1 on consent procedures.

### Analysis of youth participation

Data on PR participation in both studies was gathered through formal and informal qualitative methods, exploring experiences of both young people and adults. In both countries, data collected during the project focused on evaluation of experiences to date, feelings about tasks ahead and support needs. More in-depth evaluation at the end focused on: (1) experiences of participation, including feelings of (un)safety, (dis)respect, (lack of) appreciation, and most and least valued aspects; (2) what the project brought PRs (skills, social, results); (3) evaluation of support and training; (4) overall satisfaction and likelihood to recommend being a PR.

In Indonesia, formal evaluation of youth participation included two FGDs with PRs, one mid-way through the study, one at the end. The latter FGD also included adult staff working with the PRs. Four individual interviews were also held after the study, three with PRs and one with the adult study coordinator. All FGDs and interviews were recorded and transcribed. Participation was evaluated informally through weekly group Skype calls between the study coordinator and the PRs and regular calls between Rutgers and the study coordinator. Rutgers staff also held informal conversations with PRs during site visits and via the WhatsApp mobile messaging platform. Observations and quotes from these informal interactions were recorded in meeting notes and reports to the donor.

Youth participation in the Dutch study was evaluated through individual interviews with all PRs at three points: at the end of the two training weekends, and at the end of the project. All interviews were recorded and transcribed. Post-training interviews were carried out at the training venue by a Rutgers team member. End-of-project interviews were carried out by a Rutgers staff member (external to the research project, a young person themself), who also recorded videos of the PRs reporting on the project. The videos were edited for a conference and social media, to disseminate the results.^§^

A thematic analysis was carried out on all qualitative data on PR participation gathered on both studies, drawing out similarities and differences between different cultural contexts. This article presents the findings of that analysis, setting out the benefits, challenges and lessons learnt regarding PR involvement in these two studies. Implications of the contrasting contextual norms on gender, sexuality and youth were considered during analysis and are further discussed in the findings section below, The substantive results of the studies are published elsewhere.^6,7,33^

**Figure:**
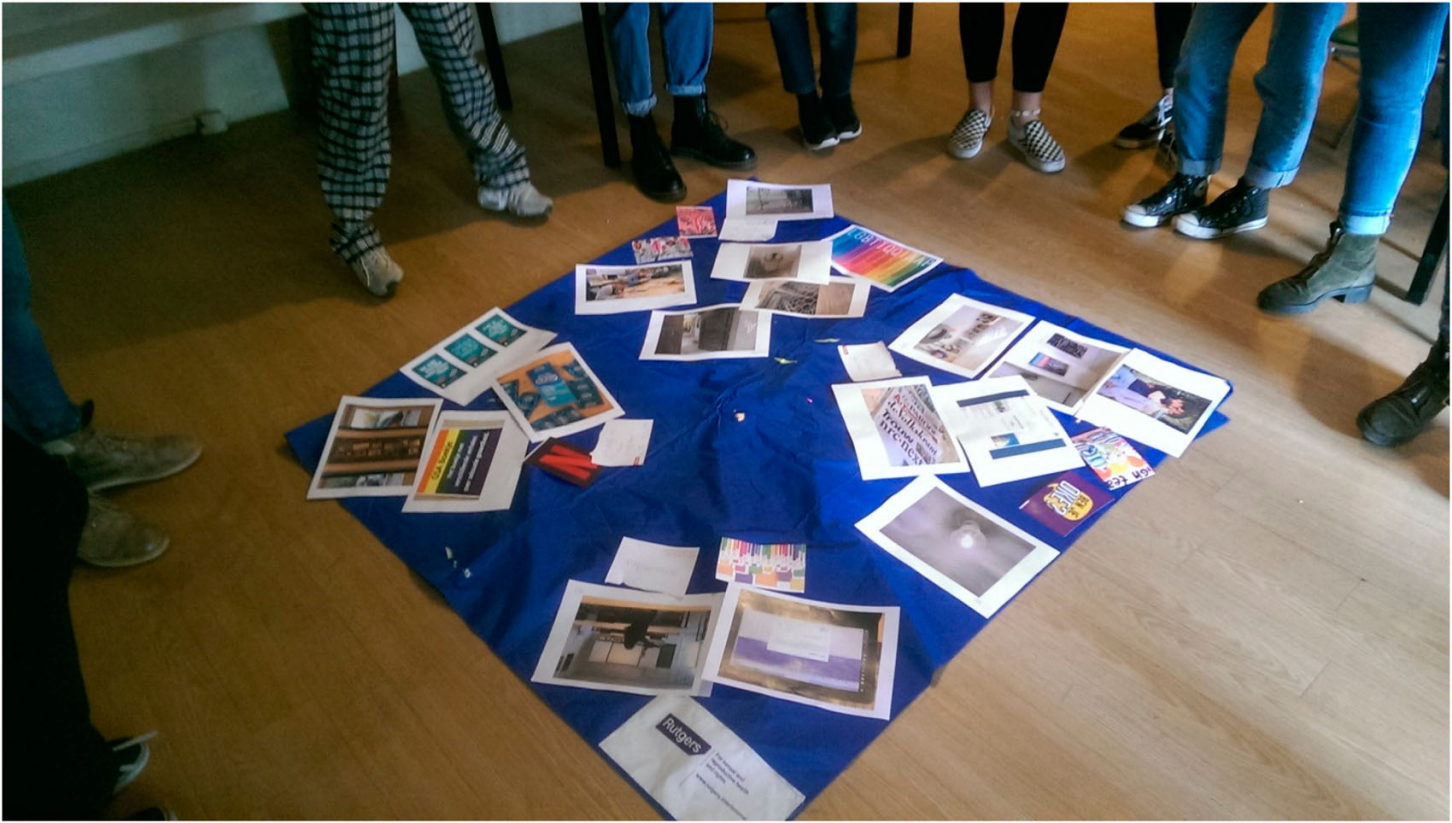
A Photovoice session during the Dutch research

## Findings

The following section sets out our findings on the benefits and challenges of involving young people as peer researchers (PRs) in sexuality studies, and the implications of these findings for future studies. This section and Table 1 summarising the findings are organised in three domains: (i) empowerment and power dynamics; (ii) taboos on sexuality and (iii) research quality and impact.

**Table 1.** Benefits, challenges and implications of involving young people as peer researchers in sexuality research

Benefits	Challenges	Implications for future studies
**Empowerment and power dynamics**		
Asserts young people’s right to participate Young people can be empowered by acquiring skills and experiences benefitting their wider lives Can deconstruct unequal power dynamics between young people and adults	Opportunities for youth empowerment can be limited by unequal youth-adult power dynamics Unequal youth-adult power dynamics may be particularly challenging in cultures of strong respect and obedience to elders	Building youth-adult partnerships is vital Different starting points of young researchers must be recognised Project leads have role to convince stakeholders of value of involving PRs Youth-adult partnerships must respond to local cultural values and age-related and broader power dynamics
**Tackling taboos on sexuality**		
Increased individual knowledge of SRHR issues. Particularly valuable in contexts of lower SRHR knowledge Rights-based approach can expand views on SRHR among individual researchers and wider community through dissemination	Gatekeepers may see it as inappropriate for young people to ask questions about sexuality Researchers may be shy in asking questions on sexuality Conflict between researchers’ personal values and project’s rights-based approach can challenge researchers and influence research processes, especially in more conservative environments	Provide support to PRs navigating potentially conflicting personal and professional (project) values Build strong relationships with gatekeepers and/or work with existing ally organisations Research commissioners should reflect on positioning, norms and values they bring to research and research design
**Research quality and ethics**		
Increased access to young people’s perspectives through an emic, insider status As young people develop experience, confidence and ownership, data quality likely increases Young people presenting findings about peers enhances credibility and impact of research dissemination	Initial data collection may be lower quality; data depth may be limited by shyness, especially where sexuality taboos stronger Youth-adult power dynamics and dominant pedagogical approaches not emphasising critical thinking may limit feasibility or depth of participatory approaches Peer researchers need additional safeguarding support when exposed to accounts of trauma and abuse Lack of academic research training may limit quality of academic style data analysis, substantiating conclusions and report writing If young people are not seen as credible researchers, research credibility may be undermined and academic rigour questioned	Quality training, ongoing support and mentoring are vital to skill development and practice. Training should critically reflect on cultural contexts, dominant pedagogical approaches and cultural norms regarding sexuality Safeguarding support to peer researchers to process accounts of trauma should include a range of options, including psychological and peer support Research designers should critically reflect on where young people most meaningfully add value to research; different roles may be suitable in different projects Public support and endorsement by authoritative senior researcher add to credibility, particularly in cultures which prioritise elder voices.

### Empowerment and power dynamics

#### Benefits: new skills and disrupting youth-adult power dynamics

A key benefit highlighted by PRs from both studies was empowerment through the acquisition of new skills. As likely in broader peer research (beyond sexuality studies), PRs reported learning new skills in qualitative research. For example, a Dutch PR said: “*Nowadays when I read about research … with interviews and focus group discussions, I think, wow, … we can do that too!”*. PRs also described gaining skills in teamwork, time management, planning, negotiation and managing competing demands, such as an Indonesian PR who explained that through involvement in the study she had: “*learned a lot … had lots of responsibility … . how to manage time … how to make a strategy … planning, stuff like that*” which she described as supporting her personal development. The studies’ focus on the sensitive issue of sexuality meant that skills such as active listening and being open and non-judgmental were particularly important. One Dutch PR reported that before starting data collection she felt uncomfortable about conducting the interviews “ *because … the topic of sexuality is a private issue … but in the end I learned a lot from interviewing, I learned to be more open.”*

Importantly, the acquisition of new skills was seen as valuable beyond the study. One Indonesian PR reported that active listening skills had “*become ingrained in us. We don’t just listen when we’re conducting our study, this becomes a habit even beyond this [research]”.* PRs from both countries said the acquisition of new skills and experiences had boosted their self-confidence, in particular with regard to employability. For the Dutch team who were high school students, new skills were seen to be particularly valuable in enhancing their CVs. For example, one Dutch PR described being a PR as “*a* *great* *experience”* which “*looks nice on your* *CV,* *you* *will have extras* *on* *top* *of* *the* *rest”.* The Indonesian team was older (21-24) and already working. They commented particularly on the experience opening up new job opportunities. For example, one Indonesian PR commented: “*The more I’m doing this [research], it’s quite interesting … I’m thinking about … using this as my future career”*.

In Indonesia, the participatory methodology of working with young people as joint decision makers also had an impact beyond empowerment of individual PRs. As is widespread in Indonesia, the Site Coordinators were previously accustomed to an authoritarian style when working with junior colleagues. They found the participatory approach a new and challenging experience and spoke positively about the personal impact of working in a more flexible and supportive way. For example one Site Coordinator said: “*I learnt how to give [young] people a chance, to trust people, I really learnt to give them opportunity [and] … freedom … I learnt to be more flexible … It was like college for me”.* Relevant for peer research more broadly, the study hence served to disrupt customary youth-adult power dynamics, particularly in a context where these are more pronounced.

#### Challenges: age-related power dynamics

Despite the above, age-related power dynamics always present key challenges in any involvement of young people in research (and broader programmes). Reflecting other projects,^5,24,36^ PRs in both studies faced poor, sometimes hostile, responses from stakeholders not accustomed to working with young people in positions of power. This was considerably notable in Indonesia where some adults struggled with working with young people as full equal team members and young people struggled to challenge elders. One adult member of the Indonesian team explained: “*In our culture it’s not easy to speak or complain to older or higher-level people. … That’s why … they [PRs] don’t speak directly to the right person to find a solution or just discuss issues”*. Gatekeepers often did not take PRs seriously, necessitating senior adult staff to intervene to demonstrate respect and elicit cooperation.^¶^

#### Implications: importance of youth–adult partnerships

These projects, like previous studies,^12,37–39^ underline how building and supporting meaningful and positive youth-adult partnerships is vital to unpacking and addressing age-related power dynamics. Project leads play an important role facilitating these partnerships to create an enabling environment for PRs. Leads must ensure all understand the benefits of working with peer researchers and how this differs from working with established researchers, or what is generally regarded as “professional research and researchers”. A key challenge is that PRs are judged against the dominant adult-expert views on what constitutes good research and a good work ethic. These views are held by adult gatekeepers and stakeholders in the research, by ourselves, and by the PRs themselves. Reflections by the Indonesian adult team members illustrate how involvement in the study enabled them challenge assumed ways of working. For example, one adult staff member explained:

*“As a mentor, I am a perfectionist, to be honest. I don't even tolerate curly hair. I am a very detailed person…I had my own ways to approach the schools, but turned out [the PRs] had their own ways. Sometimes I was upset because they were…not [doing it] according to how it should be, er, what I wanted. But I tried giving them a chance and, well, it went well. So I also learned, reflected on giving people chances. … . So I tried to be more flexible. It's fine if my hair is a little curly.”*

Building youth-adult partnerships and convincing stakeholders of the value of young people carrying out research was also necessary outside the research teams, particularly in Indonesia where age-related power dynamics were culturally stronger. It was vital to understand, recognise and work within local power-related cultural values, not only age-related, but also regarding gender, ethnicity and more. For example, some male respondents deemed it inappropriate to discuss sexuality-related issues with female researchers, while a young Javanese researcher working in Bali felt they had to work harder to gain respondents’ trust as they had different cultural backgrounds.

#### Tackling taboos on sexuality

As outlined above, both studies took a positive, rights-based approach, situating sexuality as a normal part of humanity rather than a problem. This presented both benefits and challenges.

#### Benefits: increased SRHR knowledge and understanding

In line with Rutgers’ experience (e.g. van Reeuwijk and Singh, 2018)^5^, the studies provided both countries’ PRs with opportunities to build their SRHR knowledge and understanding. Increased understanding was notable in socially conservative Indonesia, where sexuality is taboo and SRHR knowledge low.^26,40^ Sexuality is rarely talked about, as one Indonesian PR explained: “*My parents never talked about sex with me. So this only happens … when we [are] about to get married.”* Involvement in the study filled such gaps. An Indonesian PR outlined how the research had “*opened my eyes, I thought ‘wow where have I been these 23 years?’, I found out so many facts [about sexuality] I hadn’t known before.”*

Both studies actively targeted diverse participants including various minority groups and sexual orientations. Exposure to diverse narratives – supported by rights-based sex-positive diversity training – increased PRs’ understanding of diversity, for example, one PR explained: “*Now I can reach out to minority groups whose voice is rarely heard, I learned a lot from them, their experience and how they feel excluded”*. A Dutch PR explained how talking to people of different sexualities “*added to my views, because I don’t know non-heterosexual people in my own environment”.* Reflecting cultural taboos, Indonesian PRs reported greater acceptance of people with stigmatised experiences including abortion or unwanted pregnancy. One Indonesian PR outlined how their views changed: “*I used to think that people who had unwanted pregnancy was at fault … because that was how my views were constructed, how I was raised back home. Through this [research] process, I became … more … relaxed, like, ‘Oh, that’s okay.’”*

For many of the Indonesian PRs, the rights-based, positive and inclusive approach to sexuality underpinning the *Youth Voices* research project was distinctly different from their own personal (more conservative) values. Describing personal development during the project, several PRs cited learning how to “*differentiate between* *[their] professional and personal values”* as a growth area. They retained personal values but were able to also accept people with values different from their own. This is illustrated by the following quote from an Indonesian PR:

*“My personal value is, I'm sorry about this, but I fully reject LGBTQ values. However, I have professional values as well, which is to accept that they also have their own personal values.…In terms of LGBT…this project really helped me see that not everyone is toxic. They're not a disease,…they have their own values,…their own perspectives, and…well, it's their right, just like I have a right to my own personal values.”*

Through *Youth Voices*, Indonesian PRs interviewed people they had never previously encountered, including transgender and disabled people. These interviews included accounts of navigating different values systems, for example a transgender woman spoke of the importance of wearing a headscarf in recognition of her Islamic religion while also processing dismissive, discriminatory judgments about her gender identity from religious influences. Exposure to such complex stories had an important impact on the Indonesian researchers, leading them to change their views on a range of issues.

Indonesian PRs also spoke positively about disseminating their expanded views and acceptance of sexuality to friends and family. One described the research as “*a jumping-off point … on how to educate my younger siblings [about sexuality]”*, which they saw as helping protect them. Another explained:

*“We talked about gender norms in [the initial training],…that really helped me become comfortable with myself…when I returned to my hometown, I collided with my own gender norms, patriarchy, and so on. After I gained this knowledge,…I became more comfortable with myself.…So, when I saw something wrong, I was able to speak up. I feel like this is such a huge change in me, the fact that I was able to speak up.”*

#### Challenges: sensitivity of sexuality as a topic

The taboo nature of sexuality presents particular challenges when related research is carried out by young people for whom it may be seen as “too sensitive” or off limits. First, some gatekeepers deemed it inappropriate for young people to ask questions about sexuality, indicating an intersection with age-related power dynamics and viewing young people as non-sexual. Secondly, asking questions on sexuality was initially awkward and embarrassing, particularly in Indonesia. Finally, in Indonesia, although the research was designed with a positive, rights-based approach to sexuality, this was at times at odds with researchers’ personal values. While the team were increasingly able to gather data objectively, as outlined below, their interpretation of findings and suggested recommendations often reflected society’s conservative values.

#### Implications: importance of value clarification

Our experiences in these studies lead us to conclude that to facilitate effective youth engagement in sexuality research, training and mentoring should specifically address “value clarification” (see also Ngutuku and Okwany).^24^ This may follow a Socratic method, interactive dialogue discovering beliefs, assumptions and arguments and eliminating contradictions, resulting in deeper understanding and shared solutions to value conflicts.^41^ This supports researchers’ ability to navigate conflicts between values of themselves, their families and the research approach. Our experience suggests that building strong relationships with gatekeepers (e.g. schools), or working with organisations with whom there is an existing relationship, can minimise resistance to young people working on sexuality.

#### Research quality and ethics

PR engagement in sexuality research operates within two challenging domains: age-related power dynamics and sexuality-related taboos. This section identifies how these interact in the research process. Each sub-section presents the benefits and challenges to research quality and highlights the implications for future (sexuality) studies.

#### Data collection

Working with PRs helped access a broad group of young people and gather in-depth data which they may not have been comfortable expressing to an older researcher. This advantage was observed by an Indonesian adult staff member: “*[The PRs] are so natural. … not judgmental. If I were the one doing the interview, it might have turned out differently, because I already had a frame [of thinking].”*

However, this benefit was only realised when initial data quality challenges were addressed. Initially, some interviewers asked superficial questions while others probed deeper. For example one Dutch PR explained: “*At* *school* *it* *was* *like:* *do* *you* *think sex education is* *important?* *Yes. And that was all”.* Some PRs were also directive, especially when participants were not forthcoming.

Nevertheless, data quality increased as PRs acquired confidence, interviewing experience, familiarity with research objectives and content, and ownership of the research process. In both countries, professional adult researchers provided supervision and coaching during early data collection, with PRs gradually taking a more independent role, identifying research respondents and adapting the interview guides. In both countries, data collected later – when researchers were more confident and participants more candid and open to sharing personal experiences – were richer, higher quality and provided more for analysis. Similar data quality patterns, in two contrasting projects with different methodological approaches, suggest that increased experience, confidence and ownership transcend the choice of methods in increasing data quality.

Several factors meant that data quality improvement was starker in Indonesia. The Indonesian study was designed to be iterative and assumed researchers would have embedded critical thinking skills. The approach was not immediately understood by the team who were more used to working in an authoritative, directive style, and whose education had sparsely emphasised critical thinking skills. Furthermore, the Indonesian study focused explicitly on experiences of sexuality, while the Dutch study focused on the arguably more acceptable topic of sexuality education. Combined with stronger societal taboos on sexuality, this focus meant Indonesian teams had to adjust more and overcome more embarrassment in interviewing.

Experiences in both countries underline the importance of quality training and ongoing mentoring for PRs as they develop and practise data collection skills and respond to real-life challenges. Training should critically reflect on the study context, particularly dominant pedagogical approaches (indicating how accustomed researchers are to critical thinking) and cultural norms regarding sexuality.

#### Safeguarding

The explicit focus on sexuality added a safeguarding challenge in Indonesia, where PRs were exposed to first-hand accounts of (often historic) sexual harassment and abuse, posing ethical challenges to participants and researchers. Although PRs were trained how to respond to disclosure of such stories, including referral mechanisms, accounts of abuse often shocked PRs and some struggled with responses. PRs needed coaching throughout data collection on ethical participant interactions, particularly on managing expectations of researchers’ ability to help.

Exposure to these accounts also traumatised PRs. In response to this, Rutgers and UGM offered psychological support to all team members. However, uptake was low, perhaps partly due to stigma regarding seeking mental health support. Researchers preferred discussing issues within their teams and described debriefs with trusted (older) colleagues as the most helpful. Learning from this experience, we recognise the importance of tailoring support structures not only to the cultural context but further to the age and personal background of researchers (and respondents) needing support.

In the Netherlands, no traumatic stories were shared in data collection. However, researchers acquired new perspectives on their peers’ sexual identities and experiences. These insights sometimes shocked PRs and prompted them to practise taking a non-judgmental stance. These experiences underline that, regardless of context, meaningful engagement of PRs should include training and context-tailored support structures that prepare them to respond to disclosures of traumatic events, helping prevent “secondary harm”.

#### Data analysis and report writing

Both studies aimed to involve PRs in the full research process, including design, data collection, analysis and write up. To a large extent this was achieved; however, specific challenges arose in the analysis and report writing phases as insufficient consideration was given to the most inclusive and meaningful approach to involving the PRs. In Indonesia, training was provided on computer-assisted qualitative analysis, and then teams were tasked with independent analysis and report writing. However, considerably more assistance than anticipated was needed on in-depth analysis, substantiating conclusions, and report writing. This ended up taking power away from rather than giving power to the PRs. On reflection, a more supported, youth-friendly approach allowing PRs to share their perspectives but not setting unreasonably high expectations of “academic” analysis would have been more meaningful and may have yielded better results. For instance one could employ a brainstorm on findings followed by a verification of data analysis, as used successfully in studies such as those by van Reeuwijk and Singh^5^ and Singh, Both and Philpott.^2^

In the Netherlands, the PRs each analysed data they had collected and wrote their own report as a requirement for their school assignment. Training was provided by Rutgers staff to develop PRs’ analysis and report writing skills. However, analysing qualitative data proved to be quite complex for the PRs who had not previously undertaken (qualitative) research, and more ongoing support than anticipated was needed. Especially the PRs who had gathered a lot of data experienced stress in the analytical process. Rutgers researchers analysed the combined data to get an overview of the whole study. The first draft of the overall report was shared back with the PRs for their review, validation and feedback.

Experiences in both countries demonstrate that for each stage of the research, it is essential to recognise PRs’ individual starting points and critically reflect on how they can most meaningfully participate in and add value to the research and what support is needed to achieve this.

#### Research dissemination, credibility and uptake

Both projects aimed to gather evidence to make the case for sexuality education in their respective countries. PRs participated in disseminating research findings; feedback from key Dutch and Indonesian stakeholders suggests youth involvement in communicating findings powerfully increased research credibility. Findings voiced by young PRs lent credibility to claims that the research truly represented young people’s views. For example, in the Netherlands, PRs were interviewed by the host of a popular television talk show who asked: “*Do young people, in these internet times where you can find everything online, really want to talk about sex with their biology teacher?”* The PRs answered that young people want a safe space to ask questions, where their sexual and gender identities and bodily and sexual developmental differences are not stigmatised but are accepted and normalised, thus diminishing insecurities. The PRs pointed out that the internet clearly does not provide this space, making the case for comprehensive sexuality education in schools. Any answer from an adult researcher would not have been so convincing. PRs reported that visibility on national television, in newspapers, podcasts and social media strengthened their position in (school) communities, giving their views more weight.

Conversely, young people’s central role in research and dissemination led to challenges in credibility and uptake, as PRs’ academic abilities were called into question. In Indonesia, partner organisations had a strong preference that research findings be presented to local stakeholders by the Indonesian Principle Investigator, not the peer research teams, reflecting local customary respect to elders and prioritisation of voices seen as academically qualified.

#### Limitations

We note several limitations to analysis of PR participation in both studies. Data were collected by interviewers in various positions of power in relation to the PRs, which is likely to have resulted in some social desirability bias in PR responses.^42^ In both sites, interviews were conducted by staff of Rutgers, the commissioner of the broader studies. Respondents were junior team members and either project employees or volunteers. Despite the inclusive goals of both research projects, it is likely that systemic power differentials impacted the working conditions of PRs.^43^ The risk of socially desirable answers was exacerbated in Indonesia where norms on interactions with authority figures limit criticism. Echoing Bergen and Labonté,^42^ mitigation strategies for social desirability focused on building rapport with respondents, emphasising confidentiality and requesting stories to avoid generic responses. In Indonesia, data were collected by a staff member who had spent extensive time with respondents, building rapport. In the Netherlands, the appointment of a young staff member external to the study to conduct interviews aimed to reduce power differentials. Secondly, we recognise that interviewing our own colleagues and analysing our own data presents a further risk of bias.^44^

Furthermore, and perhaps most importantly, the authors recognise a limitation of the article being written on the basis of input from PRs and staff in Indonesia, but without engaging them as co-authors. Ideally, we should have engaged PRs from both sites and Indonesian colleagues in the writing of the paper. However this was not the case. We had limited time and resources to write the article, making meaningful engagement challenging. In addition, most PRs and Indonesian staff were no longer involved in the projects or employed by/engaged with the organisations/schools involved, presenting an extra barrier. Furthermore, for the Indonesian study, additional logistical challenges were presented by a language barrier with peer researchers who mostly are not confident communicating in English. Finally, engaging the PRs as co-authors would expose their identity, which might enhance social desirability bias further and/or lead them to present their data differently. Learning from this experience for future papers that evaluate youth participation or youth partnerships, we recommend considering and discussing risks for biases and repercussions versus the benefits of co-authorship with the young peer researchers, as well as planning for time and resources to enable meaningful co-authorship.

### Discussion and conclusion

Despite contrasting cultural settings, these two experiences of involving young people in sexuality research demonstrate striking similarities in challenges and factors for success. Three overarching conclusions are drawn from both sites, reflecting on the broader concept of involving young people as PRs in sexuality-related studies.

First, both projects underline the importance of training, capacity strengthening and support for individual PRs, recognising their different starting points, capacities and levels of experience. Training must recognise PRs’ specific educational backgrounds, previous work experience and cultural backgrounds as well as the research context. Attention should be paid to levels of previous education in critical thinking. Coaching is needed throughout the process, recognising that young people need time to practise and embed new skills.^4,9,18^ Expectations must be managed on how data quality and timelines may differ from projects involving older, more experienced researchers. Comparing research in different contexts illustrated the importance of considering the cultural context, including youth-adult power dynamics and norms and values regarding sexuality and age-based competencies. PRs should be supported in unpacking how their personal values may differ from the values of the study – in this case a positive, rights-based approach to sexuality. Crucially, truly meaningful involvement of young people in research requires allocating sufficient resources to allow comfortably for capacity strengthening, coaching and possibly extended timeframes; it is not a cheap fix.

Second, attention must be paid to creating an enabling environment where young people can meaningfully participate. As highlighted by others,^18,45,46^ the projects illustrate how young people can be seen as incompetent or unsuitable for involvement in sexuality research. Effective youth-adult partnerships are vital to challenge these assumptions, including dominant adult-expert views on what good research is, and with supportive management and flexible adults, willing to work differently and accept young people as equal team members. Strong youth-adult partnerships must recognise the complexities young people navigate: age-related power dynamics limiting expectations of young people’s ability; cultural taboos regarding sexuality; and the macro cultural spaces for young people’s manoeuvring.

Finally, consideration must be given to the ways young people can most meaningfully be involved in each part of the research process. This is important for research quality as well as young people’s self-esteem and confidence. In the projects explored here, young people’s role in data collection contributed to greater participant access and depth of information that may not have been achieved by older adult researchers. Yet PR involvement in data analysis was designed from an overly academic, adult-centred stance. Future research designs may adopt a more youth-friendly approach similar to van Reeuwijk and Singh,^5^ where young people shared perspectives and verified emerging conclusions rather than carrying out more rigid academic analysis.

The varied involvement of PRs in different parts of the study process reflects assertions by Treseder^47^ that different forms of youth participation may be appropriate depending on the situation, contrasting with other hierarchised models placing youth-led participation as the ideal. Treseder proposes a “degrees of participation” model of five unique but equal forms of participation. However, a considered approach is needed to ensure young people’s involvement as researchers is not tokenistic but integrated throughout the project, albeit in varying forms. Young people’s involvement in the dissemination of results can increase research credibility while concurrently strengthening their ownership of the research.

The two studies have demonstrated how engaging young people as PRs in sexuality research offers valuable opportunities for young people’s empowerment and deeper, richer research. However, these can only be achieved if sufficient attention is paid to the complex dynamics of youth-adult power relations and deep-seated taboos on sexuality, which are inextricably linked to cultural context. With the increasing shift to more youth engagement in participatory research, we underline the need to share lessons on how to manage and plan for the “sticky sides” of working with young people as peer researchers.

## Footnotes

* This paper refers to the first of two phases of *Youth Voices*. A second phase^29^ carried out later focused on respondents aged 12-13. Reports from both phases are available at https://rutgers.international/programmes/explore4action/resources/

† UGM project-managed *Youth Voices* including data collection, analysis and report writing; Rutgers delivered research design, training and technical support, and approved research outputs; PKBI hosted research teams and facilitated respondent and local stakeholder access.

‡ Photovoice is a method by which participants take photographs in response to a research question. The photographs are analysed by the same participants who work in small groups to identify common themes.

§ The resulting video, entitled ‘A message to the Minister of Education Culture and Science, Arie Slob’ can be viewed online at: https://www.youtube.com/watch?v=fBQ5IaNQeOY

¶ Where needed, these interventions were made by senior staff from Indonesian partners UGM or PKBI, or by staff from the Rutgers Indonesia office. Dutch researchers were not involved in these interventions in order to preserve local working relationships and in recognition that involvement in such situations could exacerbate challenges of power dynamics, especially in light of colonial history.
